# Efficient Explainable Models for Alzheimer’s Disease Classification with Feature Selection and Data Balancing Approach Using Ensemble Learning

**DOI:** 10.3390/diagnostics14242770

**Published:** 2024-12-10

**Authors:** Yogita Dubey, Aditya Bhongade, Prachi Palsodkar, Punit Fulzele

**Affiliations:** 1 Department of Electronics and Telecommunication, Yeshwantrao Chavan College of Engineering, Nagpur 441110, India; adityabhongade24@gmail.com; 2 Department of Electronics Engineering, Yeshwantrao Chavan College of Engineering, Nagpur 441110, India; prachi.palsodkar@gmail.com; 3 Directorate of Research and Innovation, SPDC, Datta Meghe Institute of Higher Education and Research, Wardha 442001, India; punitr007@gmail.com

**Keywords:** Alzheimer’s disease, feature selection, ensemble learning, classification, explainable AI, quantitative evaluation

## Abstract

**Background:** Alzheimer’s disease (AD) is a progressive neurodegenerative disorder and is the most common cause of dementia. Early diagnosis of Alzheimer’s disease is critical for better management and treatment outcomes, but it remains a challenging task due to the complex nature of the disease. Clinical data, including a range of cognitive, functional, and demographic variables, play a crucial role in Alzheimer’s disease classification. Also, challenges such as data imbalance and high-dimensional feature sets often hinder model performance. **Objective:** This paper aims to propose a computationally efficient, reliable, and transparent machine learning-based framework for the classification of Alzheimer’s disease patients. This framework is interpretable and helps medical practitioners learn complex patterns in patients. **Method:** This study addresses these issues by employing boosting algorithms, for enhanced classification accuracy. To mitigate data imbalance, a random sampling technique is applied, ensuring a balanced representation of Alzheimer’s and healthy cases. Extensive feature analysis was conducted to identify the most impactful clinical features followed by feature reduction techniques to focus on the most informative clinical features, reducing model complexity and overfitting risks. Explainable AI tools, such as SHAP, LIME, ALE, and ELI5 are integrated to provide transparency into the model’s decision-making process, highlighting key features influencing the classification and allowing clinicians to understand and trust the key features driving the predictions. **Results:** This approach results in a robust, interpretable, and clinically relevant framework for Alzheimer’s disease diagnosis. The proposed approach achieved the best accuracy of 95%, demonstrating its effectiveness and potential for reliable early diagnosis of Alzheimer’s disease. **Conclusions:** This study demonstrates that integrating ensemble learning algorithms and explainable AI, while using a balanced dataset with feature selection, improves quantitative results and interpretability. This approach offers a promising method for early and better-informed clinical decisions.

## 1. Introduction

Alzheimer’s disease (AD) is a significant global health concern, influencing more than 55 million people around the globe, with forecasts showing that the figure will climb to 82 million by 2030 and 138 million by 2050, according to the World Alzheimer Report 2022 [1]. The disease typically affects those aged 65 and over, providing a rising medical, social, and economic burden.

In recent years, machine learning (ML) and deep learning (DL) methods have shown great promise, especially in computer vision applications requiring high-dimensional data processing. These sophisticated models have become significant gadgets in the medical sector for Alzheimer’s disease diagnosis, prognosis, and patient outcome prediction, demonstrating their potential for transforming patient care and treatment planning [2].

The ADNI and OASIS datasets are widely used in machine learning and deep learning studies, primarily providing longitudinal and cross-sectional MRI data. Additionally, over 15% of research in this field utilizes Kaggle datasets, reflecting their growing popularity among researchers.

The success of ML and DL methods largely depends on effective feature selection. According to [3,4], biomarker genes can be identified using various feature selection techniques. The cognitive classification of dementia is closely linked to brain pathology. Research in [5] suggests that 22 neuropathological features are independently associated with dementia, with tau-related assessments being the most informative for ML classifiers. Sudharsan and Thailambal [6] have used Principal Component Analysis (PCA) for feature selection methodology and applied Regularized Extreme Learning showing significant improvement in classification strategy. Despite progress, issues persist in hyperparameter tuning and model generalizability across various populations. Much research identified the high dimensionality of data as a serious challenge, needing good feature selection strategies to improve model performance [7,8].

In the literature, machine learning (ML) has been effectively applied using a range of algorithms for Alzheimer’s research. Techniques such as Support Vector Machine (SVM), Random Forest, and various ensemble methods, including Boosting and Bagging algorithms like Gradient Boosting, XGBoost, Histogram Gradient Boosting, and AdaBoost, have demonstrated significant impact [9,10,11,12,13,14,15,16,17]. These classifiers have shown promising results in studies, highlighting their potential in improving diagnostic accuracy and disease classification. To enhance the capability of ML, methods like SHapley Additive exPlanations (SHAP) and Local Interpretable Model-Agnostic Explanations (LIME) can help make machine learning models easier to understand and work with.

A thorough evaluation of 100 research publications published between 2019 and 2022 found that 76% used deep learning (DL) methodologies. Convolutional Neural Networks (CNNs) attained the highest accuracy of 100% in categorizing AD vs. cognitively normal (CN) participants, whereas Support Vector Machines (SVMs) achieved a maximum accuracy of 99.82% [18]. Researchers have used explainable AI techniques to improve model transparency. Ref. [19] research underlines the importance of explainability in machine learning models. Using a dataset of over 169,000 records, Support Vector Machine (SVM) models performed well in identifying individuals at various stages of Alzheimer’s disease, with over 96% accuracy in separating normal cognition from Alzheimer’s. Integrating LIME and SHAP into machine learning workflows improves openness and confidence in AI systems, particularly in vital domains such as healthcare. While LIME provides localized insights appropriate for smaller models, SHAP excels at offering complete explanations required to comprehend complicated model behaviors [20,21,22]. Ongoing research investigates their applications in a variety of fields, including Alzheimer’s disease, where interpretability is critical for good clinical decision-making [23]. XGBoost is used in combination with SHAP to address class imbalance in Alzheimer’s disease (AD) progression with superior classification performance [24,25]. The literature demonstrates that applying explainable AI for effective feature identification significantly enhances model performance when combined with Bagging and Boosting classifiers. These approaches have led to remarkable improvements in the accuracy and interpretability of machine learning models [26,27,28,29,30,31]. An analysis of 23 research studies on LIME and SHAP for Alzheimer’s disease classification found that these frameworks have the potential to increase the interpretability of AI models in AD detection and classification [32,33,34]. However, it emphasizes the importance of ongoing research and development to address the obstacles and limits associated with these methodologies. While Islam et al. (2020) investigate the use of LIME for interpreting machine learning models in AD classification, Arrieta et al. (2020) classify several XAI approaches. Islam et al. particularly apply the general framework provided by Arrieta et al. to the diagnosis of Alzheimer’s disease [35,36]. Lundberg et al. expand on this concept, applying SHAP to tree-based models to enhance both local explanations and global understanding, thereby improving interpretability in machine learning applications [37,38]. Liu et al. (2024) and Rabiee et al. (2024) emphasize that comprehending model predictions is essential for clinical applications by combining SHAP with data augmentation strategies to improve the interpretability of AD classification models [39,40]. Apley and Zhu (2020) provide a comprehensive framework for visualizing the effects of predictor variables in black box models. This work not only contributes to the advancement of explainable AI but also emphasizes the importance of transparency in model predictions [41]. Ghahraman and Tamimy’s paper plays a significant role in making conversation analysis accessible and relevant to new learners using the ELI5 library for feature explanation [42]. The integration of Explainable AI techniques, such as ELI5, LIME, and SHAP, significantly enhances the transparency and interpretability of machine learning models, especially in critical domains like AD classification.

Despite significant advancements in Alzheimer’s disease (AD) classification, challenges persist in improving predictive accuracy and model interpretability, particularly when dealing with imbalanced clinical datasets and high-dimensional feature spaces. Many existing models struggle with biased predictions due to the underrepresentation of one class and often lack transparency, making it difficult for clinicians to trust the outcomes. Additionally, limited studies have focused on extensive feature analysis and reduction, critical to minimizing overfitting and enhancing model efficiency. The major contribution of the proposed framework is:

- Implementation of random sampling to mitigate the effects of data imbalance.
- Exhaustive feature analysis to identify the most relevant clinical indicators and apply feature reduction techniques to streamline the model.
- Ensemble machine learning for enhanced performance in Alzheimer’s disease classification, distinguishing between Alzheimer’s and non-Alzheimer’s cases (binary classification).
- Integration of explainable AI tools SHAP, LIME, ALE, and ELI5 to ensure that the model’s decisions are transparent and clinically interpretable.
- Evaluation of ML models based on the training time complexities.

## 2. Materials and Methods

**Figure 1:**
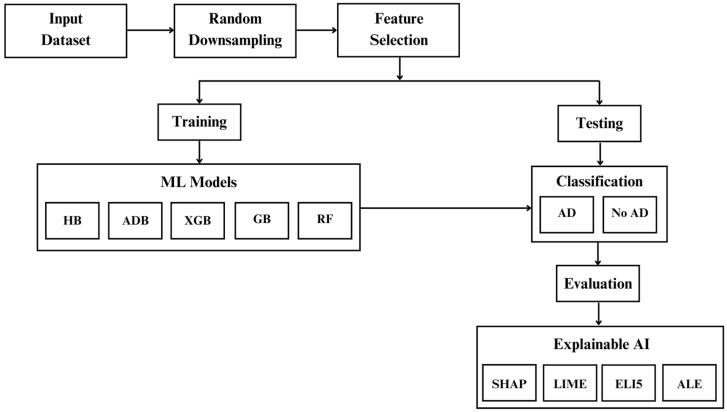
Proposed Framework for Alzheimer’s Disease Classification.

Figure 1 illustrates the workflow of the proposed method. The first step is data collection or acquiring the input dataset, followed by random sampling and feature selection. The models are trained on the training set after feature selection. After training, the models are tested using the test set for classification as AD and No AD. The test set results are evaluated quantitatively. Finally, the models are illustrated using explainable AI techniques such as SHAP, LIME, ALE, and ELI5.

### 2.1. Input Dataset

**Figure 2:**
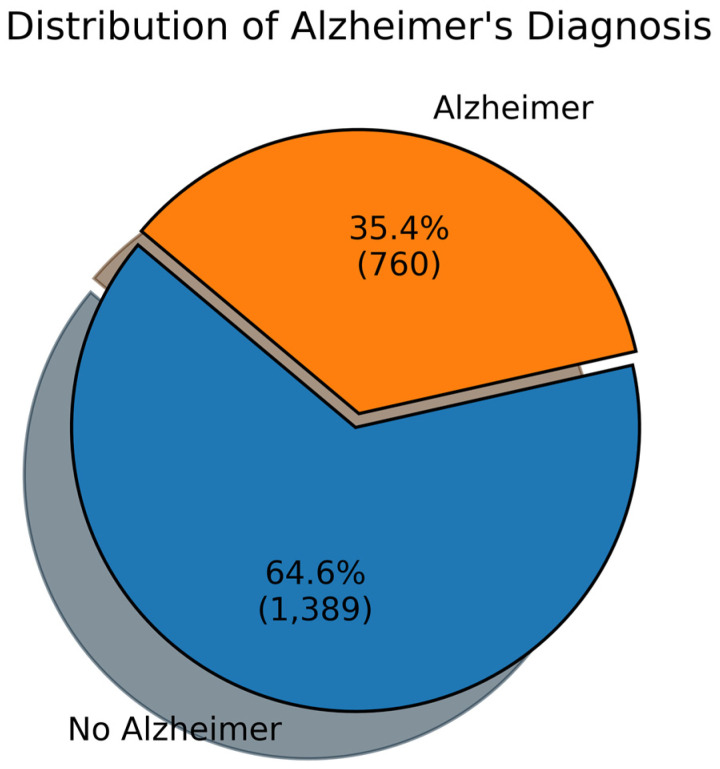
Distribution of the dataset based on diagnosis.

The dataset is sourced from Kaggle [43]. It is a publicly available dataset containing medical data of 2149 patients. It has enough data for Alzheimer’s (AD) and no Alzheimer’s (No AD) cases. Figure 2 shows the distribution of Alzheimer’s (AD) and No Alzheimer’s (No AD) cases. It can be observed that 35.6% of the entries are AD and 64.4% are no AD. It means that the majority of the entries are no AD.

The dataset contains a total of 34 input features and one output indicating the diagnosis of Alzheimer’s disease (AD), coded as 0 (No AD) and 1 (AD). These features are broadly classified into several categories: demographic factors, lifestyle factors, medical history, clinical measurements, cognitive and functional assessments, and symptoms. Out of the 34 input features, two—patient ID and doctor in charge—were removed from the experimentation. Of the remaining 32 features, 15 have numerical values. The details of these features, including their mean values, minimum values, and maximum values, are shown in Table 1. The remaining 17 features out of 32 features are categorical. The details of these categorical features are given in Table 2. The correlation of these features with the output class is calculated using the Pearson coefficient. These correlation values are also given in Table 1 and Table 2.

The age of the patients in the dataset ranges from 60 to 90 with an average age of 75. The age has a correlation of 0.005 on Alzheimer’s disease. Lifestyle factors are BMI ranging from 15 to 40 with an average value of 28 has a correlation value of 0.026, alcohol consumption in units ranging from 0 to 20 units with an average of 10 units has a correlation value of 0.007, physical activity (in hours) in week ranging from 0 to 10 with an average of 5 h in a week has a correlation of 0.0059, diet quality and sleep quality score, both ranging from 0 to 10 with an average of 5 have a correlation of 0.0085 and 0.0057, respectively. Clinical Measurements, systolic BP with the range of 90 to 180 mmHg and an average BP of 135 has a correlation of 0.0016, and diastolic BP with a range of 60 to 120 and an average value of 90 mmHg has a correlation value of 0.005. The next features, Cholesterol total (Range 150–300 mg/dL and average value of 225), Cholesterol LDL (Range 50–200 mg/dL and average value of 125), Cholesterol HDL (Range 20–200 mg/dL and average value of 60) and Cholesterol Triglycerides (Range 50–400 mg/dL and average value of 228) have a correlation value with an outcome of AD as 0.006, 0.0032, 0.0043 and 0.0023, respectively. Mini-Mental State Examination Score ranges from 0 to 30 with an average value of 15 and has a correlation of −0.24. The functional assessment score and the daily living score have a range of 0 to 10 with an average score of 5 and having correlation of −0.36 and −0.33, respectively.

Categorical demographic features are gender, ethnicity broadly classified as Caucasian, African American, Asian, and other, and education level with categories as none, high school, bachelor’s, and higher with correlation values of 0.021, 0.015, and 0.044 on AD diagnosis, respectively. Smoking status is yes or no with a correlation of 0.004 with AD diagnosis. Medical history comprising family history, cardiovascular disease, diabetes, depression, head injury, and hypertension with status as yes or no and having correlation values of 0.033, 0.031, 0.032, 0.005, 0.023, and 0.035, respectively, with AD diagnosis. Memory complaints and behavioral problems with status as yes or no and correlation values of 0.31 and 0.22, respectively, with AD diagnosis. Other symptoms such as confusion, disorientation, personality changes, difficulty completing tasks, and forgetfulness also have a status as yes or no and a correlation value of −0.019, 0.025, 0.021, 0.009, and 0.0003, respectively, with AD diagnosis.

It is observed from Table 1 and Table 2 that the top 5 features with the highest magnitude of correlation with a diagnosis are the Functional Assessment (FA) Score, Activities of Daily Living (ADL) Score, Memory Complaints, Mini-Mental State Exam (MMSE) Score, and Behavioral Problems, with correlation values of −0.36, −0.33, 0.31, −0.24, and 0.22, respectively. These features have the most significant impact on the model’s output. Features with negative correlations contribute to the probability of the output being 0, i.e., No Alzheimer’s. High values of the FA score, ADL score, and MMSE score decrease the probability of the output being 1, i.e., Alzheimer’s. In contrast, features with positive correlations increase the probability of the output being 1, i.e., Alzheimer’s. Behavioral Problems and Memory Complaints are binary features with positive correlation which means that these features being high or 1 will increase the probability of the output being 1 or Alzheimer’s. The features with low magnitudes of correlation have the least impact on the model’s output. Forgetfulness, Smoking, Total Cholesterol, etc., are some features with very low magnitudes of correlation. Thus, these features contribute the least to the probability of the output being 1 or 0.

### 2.2. Feature Analysis

**Figure 3:**
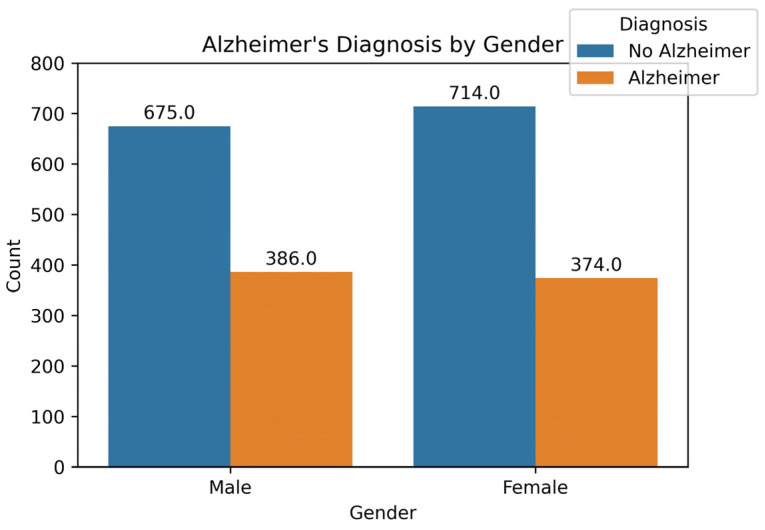
Gender-wise distribution of the dataset based on Diagnosis.

Feature analysis is carried out to find out the influence of all the features from the dataset on the outcome of the prediction task. It helps us understand the impact of a feature on the model’s output. Figure 3 shows the gender-wise count of AD and No AD cases in the dataset. There are 1061 entries labeled as male, and 1088 entries labeled as female. It can be easily calculated that the percentage of AD cases in males is (386/1061) × 100 = 36.38%, while for females, it is (374/1088) × 100 = 34.38%. The difference in the percentage of AD cases between both genders is insignificant, and gender has a very low correlation value of 0.021 with the diagnosis. This indicates that gender has little to no impact on the AD diagnosis, making it an insignificant feature in the prediction process.

**Figure 4:**
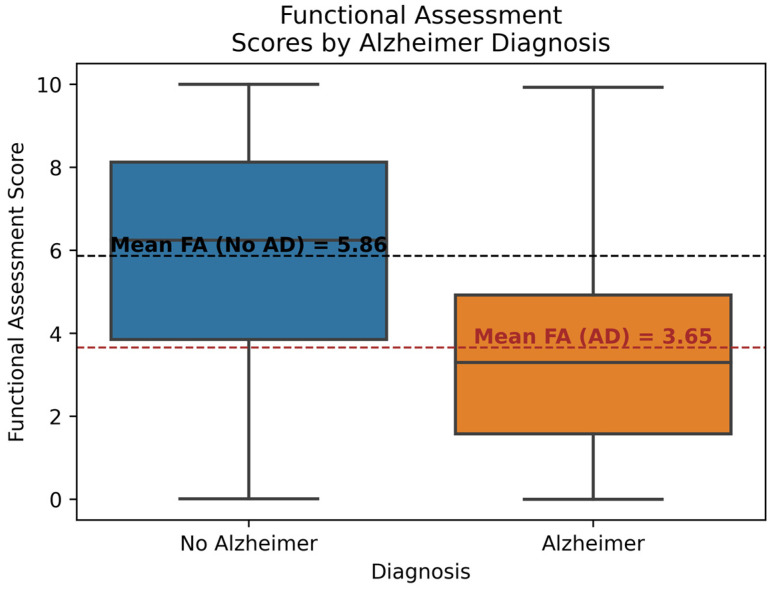
Functional Assessment Score by Diagnosis.

Figure 4 shows the relationship between Functional Assessment (FA) scores and diagnosis. The FA score is a quantitative measure of a person’s ability to perform tasks such as problem-solving, studying, social interaction, and other cognitive activities. Each box illustrates the interquartile range, minimum, maximum, and median FA scores for the corresponding group. Additionally, the mean values for both groups are highlighted. It can be observed that the mean FA score for the No AD group is 5.86, while for the AD group, it is notably lower at 3.65. Furthermore, the mean FA score of the AD group is lower than the minimum FA score of the No AD group. The FA score has a correlation of −0.36 with the diagnosis, which is considered significant. This indicates that AD patients tend to have lower FA scores, making the FA score an important feature for prediction.

**Figure 5:**
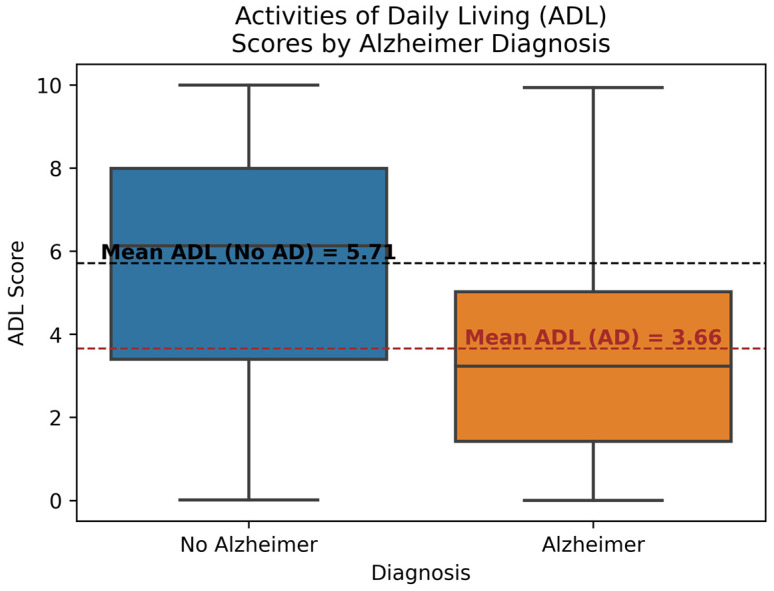
Activities of Daily Living Score by Diagnosis.

Figure 5 illustrates the differences in the ADL scores between the AD and No AD classes. ADL score refers to a quantitative measure of the ability of a person to perform daily activities like walking, eating, bathing, etc. Each box displays the interquartile range, minimum, maximum, and median ADL scores for both classes. Additionally, the mean ADL scores for both classes are highlighted. It can be observed that the mean ADL score for the No AD class is 5.71, while for the AD class, it is 3.66. The mean ADL score of the AD group is notably lower than both the mean and median ADL scores of the No AD group. The ADL score has a high correlation of −0.33 with the diagnosis. This indicates that AD patients generally tend to have lower ADL scores compared to the No AD population, making ADL scores an important feature for prediction.

**Figure 6:**
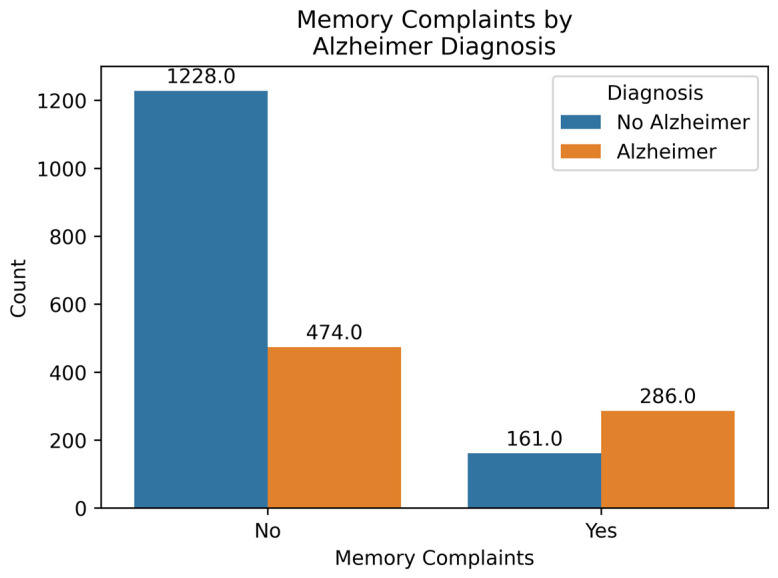
Memory Complaints by Diagnosis.

Figure 6 shows the count of AD and No AD cases in two categories: those who do not experience any memory complaints and those who do. It can be observed that the percentage of AD cases in the No Memory Complaints category is (474/1702) × 100 = 27.85%, while in the Memory Complaints category, it is (286/447) × 100 = 63.98%. The percentage of AD cases in the memory complaints category is notably higher than in the no memory complaints category. Memory complaints have a positive correlation of 0.31, indicating a significant impact on the prediction. Thus, the presence of memory complaints contributes to an increased probability of the output being 1, i.e., indicating AD.

**Figure 7:**
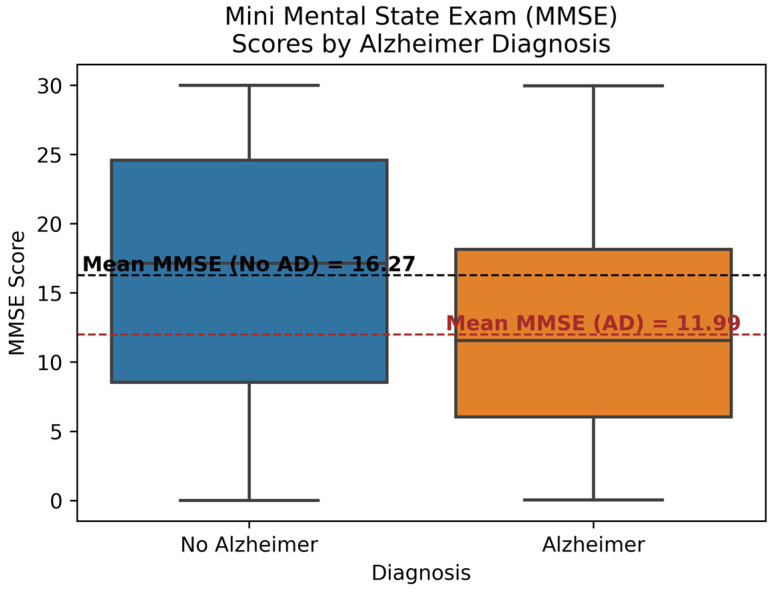
Mini Mental State Exam (MMSE) Score by Diagnosis.

Figure 7 shows the differences in MMSE scores between the AD and No AD categories. MMSE score is used to assess the cognitive and mental ability of a person. It helps to evaluate the attention, recall, orientation, etc., of a person. Each box represents the interquartile range, minimum, maximum, and median MMSE scores for the corresponding group. The mean MMSE score is also highlighted in the figure, showing that the mean for the No AD group is 16.27, while for the AD group, it is 11.99. The mean and median MMSE scores for the AD group are significantly lower than those for the No AD group. This indicates that AD patients tend to have lower MMSE scores relative to the No AD population. MMSE score also has a high correlation of −0.24, making the MMSE score an important feature in the prediction process.

**Figure 8:**
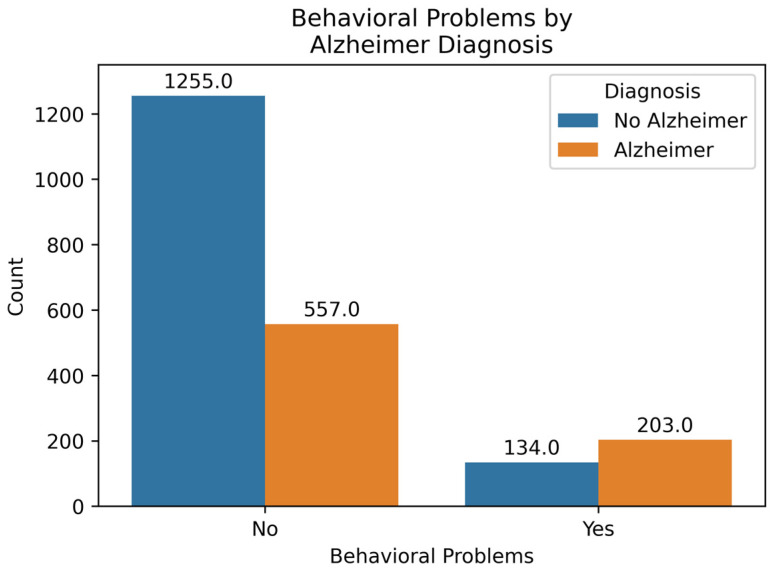
Behavioral Problems by Diagnosis.

Figure 8 shows the count of AD and No AD cases in two categories: those who do not experience any behavioral problems and those who do. The percentage of AD cases in the Behavioral Problems category is (203/337) × 100 = 60.24%, while in the No Behavioral Problems category, it is (557/1812) × 100 = 30.74%. Behavioral problems have a positive correlation of 0.22, indicating a considerable impact on the prediction. Therefore, having behavioral problems contributes to an increased probability of the output being AD.

**Figure 9:**
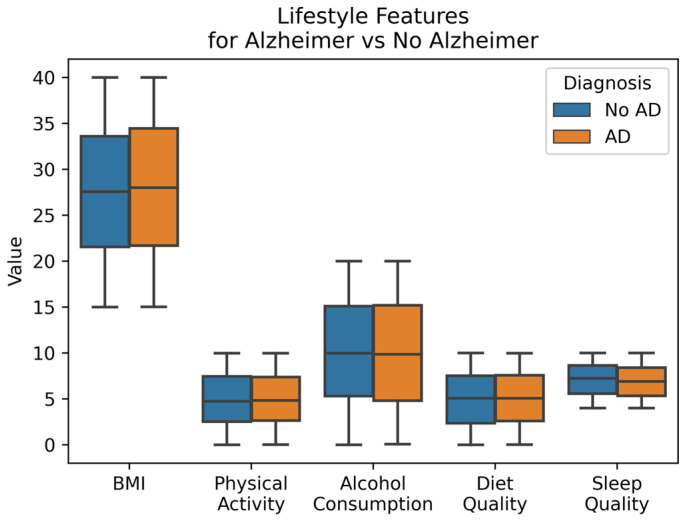
Lifestyle Features by Diagnosis.

Figure 9 shows the relationship between AD diagnosis and various lifestyle-related features, including Body Mass Index (BMI), Physical Activity, Alcohol Consumption, Diet Quality Score, and Sleep Quality Score. It can be observed that there is no significant difference in the average values of these features for both cases, AD and No AD. BMI, Physical Activity, Alcohol Consumption, Diet Quality Score, and Sleep Quality Score have correlation values of 0.026, 0.006, 0.007, 0.009, and 0.057, respectively, which are very low. This indicates that these features have a minimal impact on the prediction.

**Figure 10:**
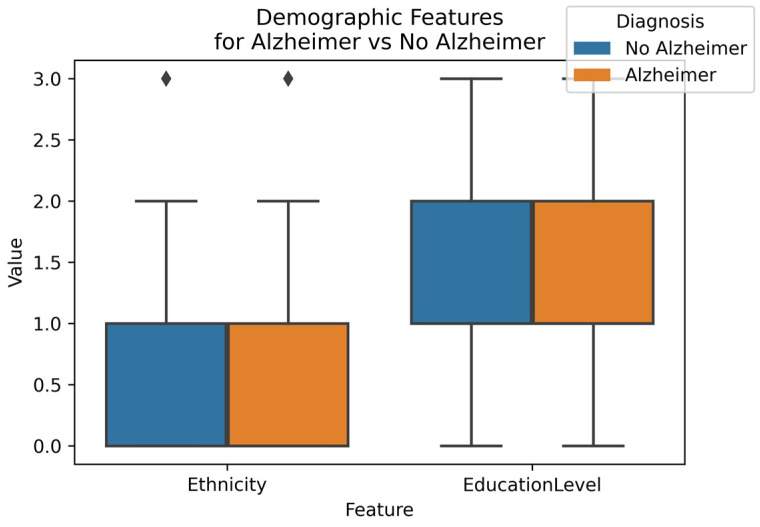
Demographics Features by Diagnosis (Diamond indicate outliers).

Figure 10 shows the relationship between AD diagnosis and the categorical demographic features, Ethnicity, and Education Level. It can be observed from the figure that the plots for both features are almost identical in both cases. Ethnicity and Education Level have correlation values of 0.015 and 0.044, respectively, with the diagnosis, indicating that they have minimal impact on the prediction. 

**Figure 11:**
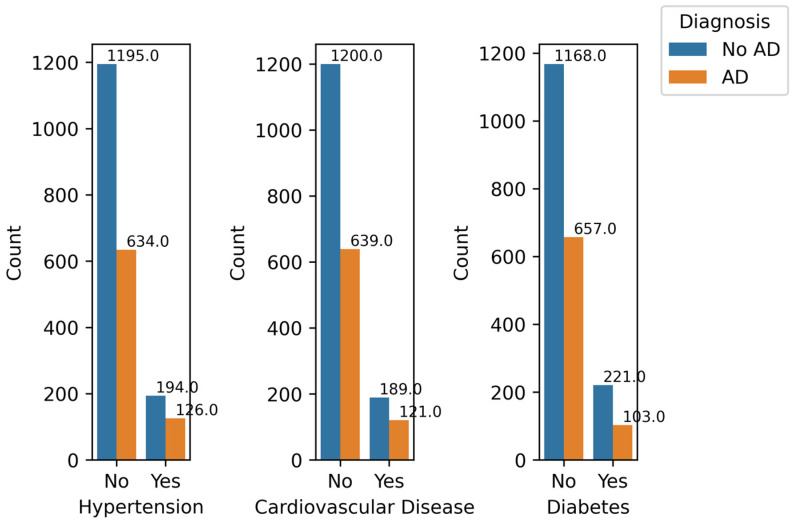
Health Conditions by Diagnosis.

Figure 11 illustrates the relationship between AD diagnosis and health conditions: Hypertension, Cardiovascular Disease, and Diabetes. It can be observed that there is no major difference in the ratios of AD cases to No AD cases for these features. Hypertension, Cardiovascular Disease, and Diabetes have correlation values of 0.035, 0.031, and 0.032, respectively, which are very low. This indicates that the presence or absence of these features has a minimal impact on the prediction.

**Figure 12:**
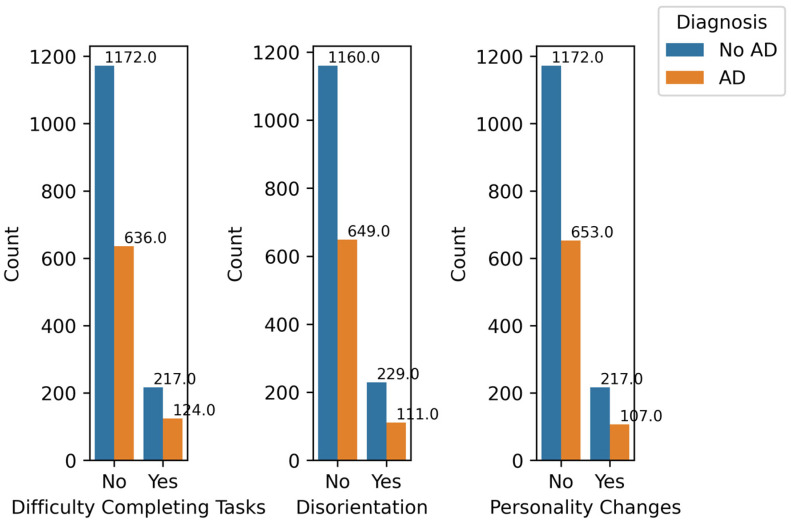
Severe Symptoms by Diagnosis.

Figure 12 shows the relationship between AD diagnosis and severe symptoms: Difficulty Completing Tasks, Disorientation, and Personality Changes. It can be observed from the figure that the differences among the ratios of AD cases to No AD cases for these features are minimal. Difficulty Completing Tasks, Disorientation, and Personality Changes have low correlation values of 0.009, 0.025, and 0.021, respectively. These values are relatively low, indicating a low impact of these features on the prediction.

**Figure 13:**
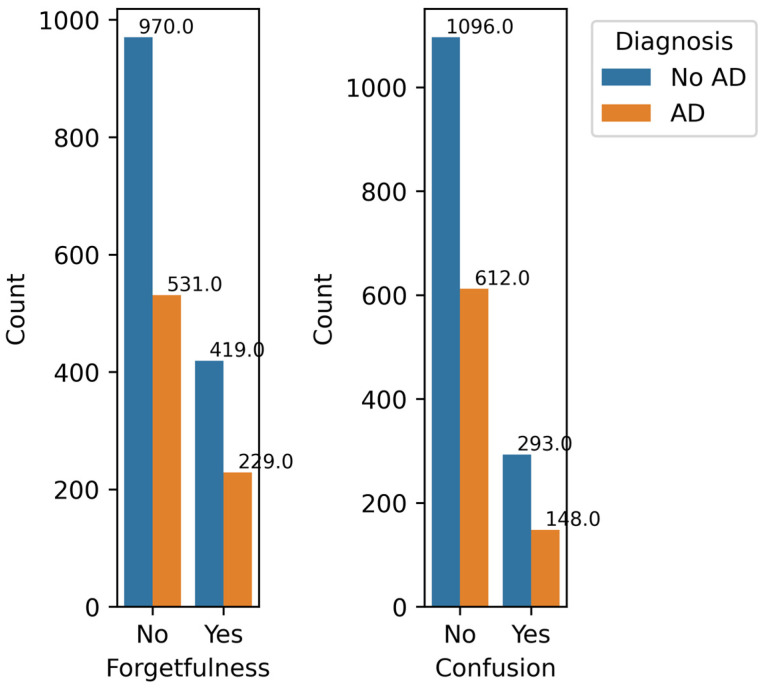
Lenient Symptoms by Diagnosis.

Figure 13 shows the count of AD and No AD cases for the absence and presence of Lenient Symptoms, Forgetfulness, and Confusion. Out of 2149 total samples, 1501 do not experience forgetfulness, while the remaining 648 do. The percentage of AD cases in the subset that does not experience forgetfulness is (531/1501) × 100 = 35.38%, and for the subset that does, it is (229/648) × 100 = 35.34%. The difference between the percentage of AD cases in both subsets is insignificant. Additionally, forgetfulness has a very low correlation value of 0.0003 with AD diagnosis, making it a feature with minimal impact.

Similarly, 1708 individuals do not experience confusion, while the remaining 441 do. The percentage of AD cases in the subset that does not experience confusion is (612/1708) × 100 = 35.83%, while for those that do, it is (148/441) × 100 = 33.56%. Like forgetfulness, confusion has a low correlation of −0.019 with AD diagnosis, making it another low-impact feature. Therefore, both forgetfulness and confusion have very little influence on the model’s prediction and can be discarded without affecting the model’s performance.

### 2.3. Random Downsampling

**Figure 14:**
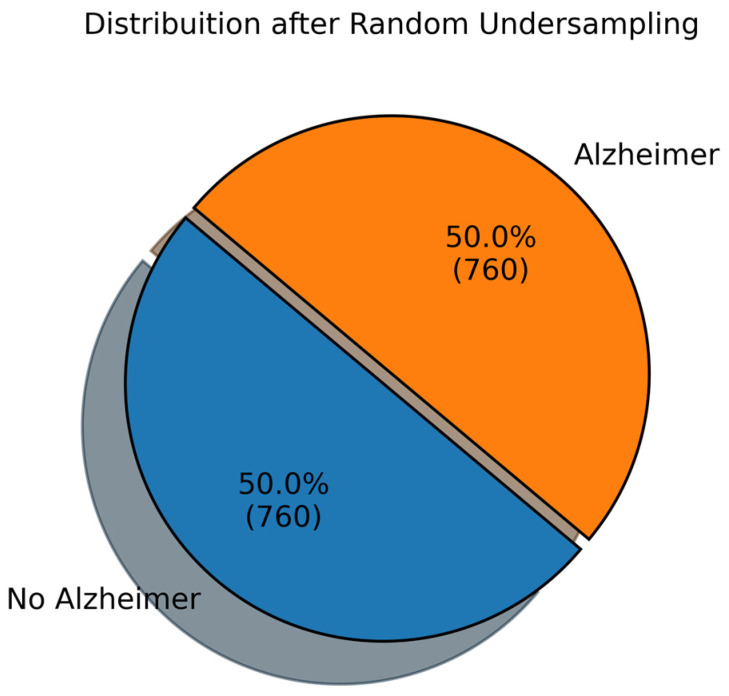
Distribution of the down-sampled dataset based on Diagnosis.

Since the majority (64.6%) of the entries are no AD, it may cause the model(s) to be biased. To ensure minimal bias, we randomly undersample the majority of entries. This means deleting or eliminating the majority or No AD entries after randomly shuffling them. This process is performed to make the distribution equal, i.e., 50% for AD and 50% for No AD. It causes the number of total entries to reduce. Random sampling is used to down sample the No AD 1389 samples to 760 AD samples to match the number of AD samples. In random sampling, each data point has an equal probability of being selected. We have total n=1389 No AD samples as $X=\left\{x_{1},x_{2},\ldots \ldots x_{1389}\right\}$ and they down sampled to the set S of size k=760. Each sample from X has a probability $\frac{1}{n}=\frac{1}{1389}$ of being selected and we continue the pass k times to form the new datasets of No AD samples as $S=\left\{s_{1},s_{2},\ldots \ldots s_{760}\right\}$. Hence, the probability that a specific sample is chosen in the set S is $p\left(x_{i}\in s\right)=\frac{k}{n}$. The distribution of data after ransom sampling is shown in Figure 14. 

### 2.4. Feature Selection

As learned above, many features have very low correlation values with the diagnosis, only a few features can be used for the prediction task without hampering the performance much. This reduces the dimensionality and computational cost significantly. The 5 best features are selected for training the models using the K-Best Method. Feature selection eliminates irrelevant features from the dataset. The K-Best Method calculates the feature score for each score and retains only top K features according to their feature scores.

The feature score is calculated to find its relevance to Alzheimer’s disease. The score is calculated using Chi-square test for categorical features and ANOVA F-value for features with continuous value and mutual information between two features. The feature vector for the Alzheimer’s dataset has a total of 32 features represented as $\left[X_{1},X_{2},\ldots \ldots X_{32}\right]$. For each feature $X_{i}$, feature score $S_{i}$ calculated using a chi-squared test $\chi^{2}=\sum \frac{{\left(O-E\right)}^{2}}{E}$ with O as observed occurrence and E as expected occurrence. F-score gives the variance between the groups relative to the variance within the groups. The feature score is calculated using $F=\frac{\mathrm{variance}\;\mathrm{between}\;\mathrm{group}}{\mathrm{variance}\;\mathrm{within}\;\mathrm{group}}=\frac{\mathrm{MSB}}{\mathrm{MSW}}$. Here, variance between group MSB is calculated using $MSB=\frac{\sum_{j=1}^{k}n_{j}{\left({\bar{x}}_{j}-\bar{x}\right)}^{2}}{k-1}$ and variance within the group MSW is calculated using $MSW=\frac{\sum_{j=1}^{k}\sum_{i=1}^{n_{j}}{\left(x_{ij}-{\bar{x}}_{j}\right)}^{2}}{n-k}$ with $n_{j}$ is the number of observations in group j, ${\bar{x}}_{j}$ is the mean of group j, $\bar{x}$ is the overall mean and $x_{ij}$ is the observation in group j. Feature score $S_{i}$ for each feature $X_{i}$ are ranked in descending order. This ranking reflects the relevance of each feature in predicting the target variable. $X_{ranked}=\left\{X_{\pi \left(1\right)},X_{\pi \left(2\right)},\ldots \ldots X_{\pi \left(32\right)}\right\}$ where π is a permutation that orders the scores $S_{i}$ in descending order.

### 2.5. Machine Learning Algorithms

Machine learning algorithms are applied on Alzheimer’s dataset for diagnosis of Alzheimer’s disease as AD and No AD. Five ensemble-based machine learning algorithms are applied, and they are discussed below.

#### 2.5.1. Random Forest (RF)

A Random Forest builds T decision trees $f_{i}$ which are trained on different random samples or bootstrapped samples $D_{i}$ of features from the training dataset. A decision tree recursively splits the data using feature values to classify the patient as AD or No AD based on information gained at each node [16,17]. The final prediction of the RF model $f_{RF}\left(X\right)$ calculated using taking the average or majority vote of the predictions from all the trees as

$$f_{RF}\left(X\right)=\frac{1}{T}\sum_{i=1}^{T}f_{i}\left(X\right)$$

where $f_{RF}\left(X\right)=\left\{0,1\right\}$ is the prediction of the $i^{th}$ tree (0 = No AD, 1 = AD) for the input features X. For classification, the output is usually the class with the majority vote as

$$f_{RF}\left(X\right)=\mathrm{mode}\left(f_{1}\left(X\right),f_{2}\left(X\right),\ldots \ldots f_{T}\left(X\right)\right)$$

#### 2.5.2. Gradient Boosting

Gradient boosting is used for Alzheimer’s classification where the model builds a strong classifier by combining many weak classifiers or trees. This model minimizes a loss function using gradient descent in function space, with each new tree helping to reduce the classification error on the training data. This approach leads to a highly accurate model and is well suited for diagnosing Alzheimer’s disease [9,15]. The steps involved are described below.

The objective function $L\left(y,\hat{y}\right)$ measures the difference between the actual label $y\;\left(0,1\right)$ and the predicted label $\hat{y}$. Gradient boosting builds the model in an additive manner by sequentially adding weak learners. Each weak learner is trained to correct the errors of its predecessor. The model at $m^{th}$ iteration is updated as $F_{m}\left(x\right)=F_{m-1}\left(x\right)+\eta h_{m}\left(x\right)$. Here $F_{m-1}\left(x\right)$ is the model at the previous iteration, η is the learning parameter, $h_{m}\left(x\right)$ is the new weak learner added at iteration calculated using

$$h_{x}\left(x\right)=\arg\;\underset{h}{\min}\sum_{i=1}^{n}{\left({\tilde{r}}_{i,m}-h\left(x_{i}\right)\right)}^{2}$$

where ${\tilde{r}}_{i,m}$ is the residual for the $i^{th}$ data point at iteration, which represents the direction and magnitude of the error and $h\left(x_{i}\right)$ is the prediction of the weak learner for the $i^{th}$ data point.

#### 2.5.3. Extreme Gradient Boosting (XGB)

XGB includes $L_{2}$ and $L_{1}$ regularization to control model complexity and also avoid overfitting. The regularization in XGB is $\Omega \left(f_{k}\right)=\gamma T+\frac{1}{2}\lambda \sum_{j=1}^{T}w_{j}^{2}$ with γ and λ are the hyperparameter that controls regularization strength.

#### 2.5.4. AdaBoost Gradient Boosting (ADB)

ADB constructs a strong classifier by iteratively improving the performance of weak classifiers. Each weak classifier is trained on the data, and AdaBoost focuses more on the misclassified samples in subsequent rounds, adjusting their weights to prioritize harder-to-classify cases. The weights are initialized for each of n sample as $w_{i}^{\left(1\right)}=\frac{1}{n},\;for\;i=1,2,\ldots n$. Weak learners are trained at each iteration and then weighted error $\varepsilon_{t}$ is calculated to determine how many samples are misclassified. The weight $\alpha_{t}$ of the weak classifier $h_{t}$. Finally, the weights are updated using $w_{i}^{\left(t+1\right)}=w_{i}^{\left(t\right)}.e^{\alpha_{t}1\left(h_{t}\left(x_{i}\right)\neq y_{i}\right)}$ [12].

#### 2.5.5. Histogram Boosting (HB)

HB groups continuous features into grouped into a fixed number of discrete values, which reduces computation. Instead of evaluating every unique feature value for splitting, the algorithm evaluates bins [3]. After that, decision trees are constructed by finding splits based on histogram bins, which represent ranges of feature values. This speeds up the tree-building process in each iteration. At each iteration, the model improves by fitting a weak learner to the residual errors of the previous model. The final model is a weighted sum of all weak learners computed as $f\left(x_{new}\right)=\sum_{t=1}^{T}\eta .h^{\left(t\right)}\left(x_{new}\right)$. The boosting process then iteratively minimizes a classification loss function $Loss=-\sum_{i=1}^{N}\left(y_{i}\log\left(p_{i}\right)+\left(1-y_{i}\right)\log\left(1-p_{i}\right)\right)$ for Alzheimer’s disease classification [14].

### 2.6. Explainable AI

This section describes the explainable model used for model interpretability. Various interpretable models described in the literature are reported in this section, like LIME, SHAP, ALE, and ELI5. XAI improves model transparency, visualizing the effects of predictor variables, which plays a significant role in making conversation analysis accessible and relevant to new learners for feature explanation. Healthcare and clinical analysis become more authentic and interpretable.

#### 2.6.1. Local Interpretable Model-Agnostic Explanations (LIME)

LIME is a model-agnostic interpretability technique used to explain the predictions of any machine learning model. LIME provides a local, interpretable linear model to approximate the model f decision-making process around the input feature x. LIME first generates a neighborhood of data points around the instance x by perturbing the feature values. Let z be the set of perturbed samples. Each $z_{i}\in Z$ is created by slightly altering the feature values of x simulating variations in clinical measurements. For the input feature $z_{i}\in Z$ the perturbed samples are $z_{i}=x+\varepsilon_{i}$. The model f is used to predict the outcome of each perturbed data point with probability $f\left(z_{i}\right)$. LIME assigns a weight to each perturbed instance based on its similarity to the original instance x. The similarity is measured using a Euclidean distance $d\left(x,z_{i}\right)$ or any kernel function $\pi_{x}\left(z_{i}\right)=\exp\left(-\frac{d\left(x,z_{i}\right)}{\sigma^{2}}\right)$ and σ controls the spread of the kernel. Using the weighted perturbed samples Z and their corresponding predictions from f, LIME fits a simple, interpretable linear model (e.g., a weighted linear regression) to approximate locally around x.

The interpretable model $g\left(z\right)=\beta_{0}+\beta_{1}z_{1}+\ldots \ldots \beta_{n}z_{n}$ where $\beta_{0},\beta_{1},\ldots \ldots \beta_{n}$ are the coefficients that explain how each clinical feature contributes to the local prediction. The coefficients $\beta_{i}$ are learned by minimizing a weighted loss function $\underset{\beta }{\min}\sum_{i}\pi_{x}\left(z_{i}\right){\left(f\left(z_{i}\right)-g\left(z_{i}\right)\right)}^{2}$. For Alzheimer’s classification, the feature with the largest positive would be the most influential in predicting Alzheimer’s, while a negative $\beta_{i}$ would suggest a feature pushing the prediction away from the Alzheimer’s class [36,37].

#### 2.6.2. Accumulated Local Effects (ALE)

ALE plots are used to interpret the behavior of machine learning models by estimating how individual features influence the model’s predictions, while considering interactions between features. ALE plots estimate the average marginal effect of a feature on the model prediction, but locally and accumulated over the feature’s domain [41]. For a clinical feature $x_{j}$ ALE quantifies the effect of changing $x_{j}$ on the model’s output as

$$ALE_{j}\left(x_{j}^{\left(k\right)}\right)=\sum_{k=1}^{K}\frac{1}{\left|D_{x_{j}^{\left(k\right)}}\right|}\sum_{x\in D_{x_{j}^{\left(k\right)}}}\left[f\left(x_{1},\ldots \ldots x_{j}^{\left(k\right)},\ldots \ldots ,x_{n}\right)-f\left(x_{1},\ldots \ldots x_{j}^{\left(k-1\right)},\ldots \ldots ,x_{n}\right)\right]$$

It accumulates these local effects across the feature’s value range, providing insights into how the clinical feature influences the model’s predictions in an interpretable manner.

#### 2.6.3. SHapley Additive exPlanations (SHAP)

SHAP provides an interpretable method to understand how clinical features influence Alzheimer’s disease classification models. It provides both global and local explanations. The SHAP value $\phi_{j}\left(x\right)$ for feature $x_{j}$ quantifies how much $x_{j}$ contributes to the difference between the model’s prediction $f\left(x\right)$ and the baseline prediction (the average prediction over all patients). It is calculated using

$$\phi_{j}\left(x\right)=\sum_{S\subset \left\{x_{1},x_{2},\ldots x_{n}\right\}\backslash \left\{x_{j}\right\}}\frac{\left|S\right|!\left(n-\left|S\right|-1\right)!}{n!}\left[f\left(S\cup \left\{x_{j}\right\}\right)-f\left(S\right)\right]$$

The prediction $f\left(x\right)$ for patient x can be decomposed as $f\left(x\right)=\phi_{0}+\sum_{j=1}^{n}\phi_{j}\left(x\right)$.

SHAP provides a complete decomposition of the model’s prediction into additive contributions from the clinical features [39,40].

#### 2.6.4. ELI5

ELI5 is a tool for making machine learning models more interpretable which uses permutation importance to interpret the model. The feature $x_{i}$ in the dataset is shuffled and a change in model performance is observed. If shuffling $x_{i}$ leads to a significant decrease in accuracy, then feature $x_{i}$ is considered important. The weight of feature $x_{i}$ is computed as $Performance_{\left(original\right)}-Performance_{shuffled}$.

$Performance_{original}$ is the model’s accuracy or another performance metric (e.g., AUC-ROC) on the unshuffled data. $Performance_{shuffled}$ is the performance when feature $x_{i}$ is shuffled.

ELI5 assigns importance to each based on how their weight $w_{i}$ affects the likelihood of classifying a patient as having Alzheimer’s disease. The final prediction will be a weighted sum of the features. ELI5 helps us understand which clinical features are most important in predicting the disease [42]. The tool provides both global and local explanations. For simple models, it uses feature weights and for complex models, it uses permutation importance to quantify how much each feature contributes to the model’s predictions.

## 3. Results

The quantitative analysis of the proposed framework for Alzheimer’s diagnosis is carried out using various metrics. The explanation of the results obtained is provided using the interpretable model. This section provides comprehensive analysis of simulation results and an explanation for Alzheimer’s disease diagnosis.

### 3.1. Quantitative Metrics

The five-ensemble machine learning (ML) algorithms used for the Alzheimer’s Disease (AD) diagnosis using the proposed framework are evaluated using various quantitative metrics. The confusion matrix is generated for each ML algorithm. TP indicate the patients with AD correctly predicted with positive AD. TN indicate patient with No AD is correctly predicted as having No AD. FP indicate patient with No AD is incorrectly predicted as positive AD and FN indicate an AD patient is incorrectly predicted as No AD. Based on these four parameters, the following metrics are calculated for quantitative assessment of the proposed framework for AD diagnosis. Proportion of correct prediction for both AD and No AD is $\mathrm{Accuracy}=\frac{\mathrm{TP}+\mathrm{TN}}{N}$. Proportion of predicted AD cases that were truly AD is $\mathrm{Precision}=\frac{\mathrm{TP}}{\mathrm{TP}+\mathrm{FP}}$. The proportion of actual AD cases that were correctly identified is $\mathrm{Recall}=\frac{\mathrm{TP}}{\mathrm{TP}+\mathrm{FN}}$. Harmonic mean of precision and recall, balancing both metrics is $F_{1}\text{-}\mathrm{Score}=2\times \frac{\mathrm{Precision}\times \mathrm{Recall}}{\mathrm{Precision}+\mathrm{Recall}}$.

Log Loss measures the accuracy of predicted probabilities by comparing them to actual outcomes (No AD or AD, in our case) given by $\mathrm{Log}\;\mathrm{Loss}=-\frac{1}{N}\left(\sum_{i=1}^{N}\left(y_{i}\log\left(p_{i}\right)+\left(1-y_{i}\right)\log\left(1-p_{i}\right)\right)\right)$, here $y_{i}$ is the actual class labels for AD for $i^{th}$ instance and $p_{i}$ is the predicted probabilities of class with AD for $i^{th}$ instance, N is the total number of instances in the dataset. It penalizes incorrect predictions proportional to their confidence levels. Specifically, the higher the confidence of an incorrect prediction, the greater the penalty incurred.

For our application, if the model predicts a high probability (e.g., 0.97) for No AD when the actual label is AD, the log loss will incur a significant penalty due to the high confidence in an incorrect prediction. Therefore, a lower log loss is desirable because it indicates that predicted probabilities are closely aligned with the actual output, signifying more accurate predictions.

### 3.2. Quantitative Evalaution

Table 3 shows the training and testing accuracies of the models used for predicting Alzheimer’s Disease (AD). It can be observed that Random Forest and XGB show perfect training accuracy (1.000), which indicates overfitting with lower testing accuracies of 0.949561 and 0.932018, respectively. AdaBoost and Histogram Gradient Boosting reported training accuracy of 0.9229 and 0.999, respectively, and relatively high testing accuracies (0.91667 and 0.9407, respectively). The lower training accuracy of AdaBoost suggests it might not be as flexible in fitting the training data compared to the other models. Gradient Boosting has a well-balanced performance, with a training accuracy of 0.956767 and the highest testing accuracy of 0.951754, suggesting good generalization.

Table 4 shows the quantitative evaluation of the ML models using the metrics of Precision, Recall, Specificity, and F1 Scores. It can be observed that GB has produced precision, recall, specificity, and F1 score values of 0.951976, 0.951754, 0.948, and 0.951792, respectively, outperforming every other model in all metrics. The high recall suggests that GB is capturing most of the true positive cases effectively and the precision indicates it has fewer false positives. RF outperforms every model except GB, with precision, recall, specificity, and F1 score values of 0.949704, 0.949561, 0.948, and 0.949591, respectively. Both GB and RF produced an equal specificity of 0.948. ADB produced the lowest precision, recall, specificity, and F1 score values of 0.921322, 0.916667, 0.88, and 0.916861, respectively, underperforming every other model used

Table 5 shows the quantitative evaluation of ML models based on the Jaccard Score (JS), Dice Coefficient (DC), and Matthews Correlation Coefficient (MCC). It can be observed that GB performs the best overall based on these metrics, with the highest JS of 0.899543, DC value of 0.947115, and MCC of 0.902909, suggesting strong performance in both precision and recall, as well as good overall correlation between predicted and actual labels. RF follows closely behind GB with a DC value of 0.944578 and MCC 0.898384. XGBoost shows slightly lower performance with JS of 0.862332 and MCC 0.863964, but it is still competitive with a decent DC value of 0.926366. AdaBoost has the lowest performance of the models evaluated, with a JS of 0.838983, a DC value of 0.912442, and a MCC of 0.83724, indicating it may not be as effective for this task. HB reported is performing better than XGBoost and AdaBoost, but slightly behind GB and RF with JS of 0.878378, DC value of 0.9352, and MCC of 0.880936.

**Figure 15:**
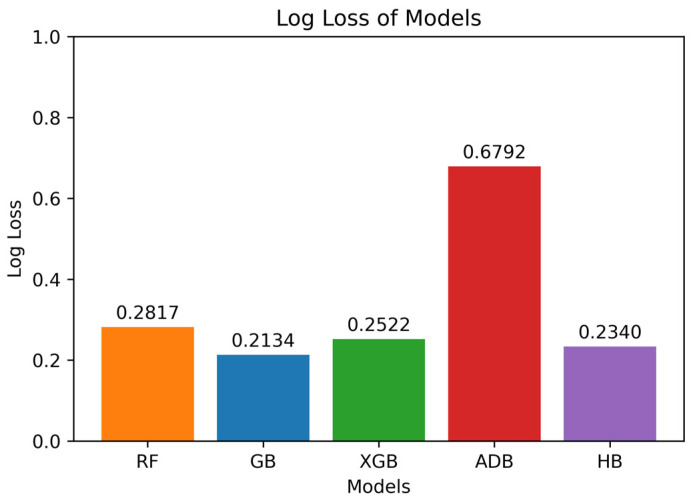
Log Loss of ML models.

Figure 15 displays the Log Loss for the models used. As discussed above, the lower the log loss, the better the model’s performance is. It can be seen that GB produced the least log loss of 0.2134 among the models used indicating that GB makes accurate predictions for AD diagnosis. HB and XGB also perform quite well, with log loss values of 0.2340 and 0.2522, respectively. In contrast, ADB produced the highest log loss value of 0.6792 among all the models, indicating that it underperformed relative to all the models. GB having the least log loss makes it reliable for the prediction task.

**Figure 16:**
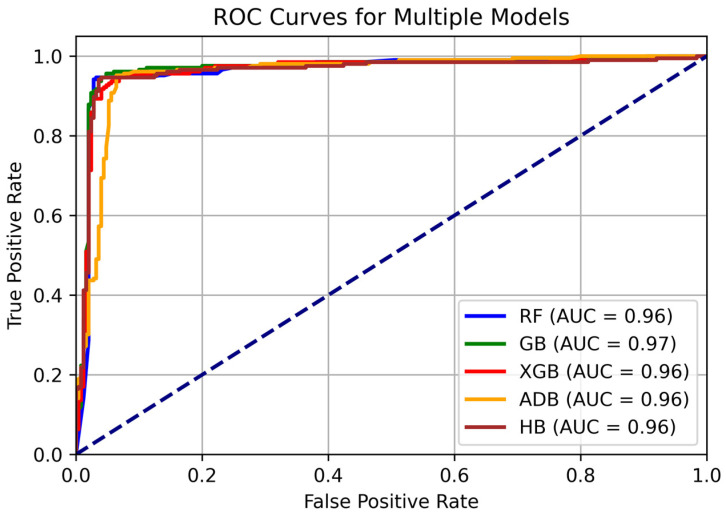
ROC Curves for the used ML models.

Figure 16 illustrates ROC curves for the ML models being used for prediction. It can be observed GB has the highest AUC value of 0.97, indicating its ability to discriminate between AD and No AD Classes. Other models also have very strong AUC values of 0.96. These models are also performing well in terms of distinguishing between AD and No AD classes.

Training time complexity refers to the measure that illustrates how the training time of a ML model grows or varies with changes in the input dataset size, i.e., the number of samples. It is used to compare the efficiency of ML models in learning from a given dataset. Time complexity is typically measured in arbitrary units; here, we have used time in seconds to measure the training time for each model.

A gradually increasing or flat curve indicates that the training time does not rise significantly with an increase in input size, making it favorable. In contrast, a steep curve shows that the training time increases sharply with even a small increase in dataset size, which is less favorable.

The training time of each model for different numbers of samples is calculated by training each model on input datasets ranging from 100 to 10,000 samples. The make classification() function from the Scikit-learn library is used to generate synthetic datasets with 20 input features and varying sizes. The time taken for each model to train on a particular dataset is recorded using the time() function from the time library. Finally, the curves are plotted for each dataset size with the corresponding training time.

**Figure 17:**
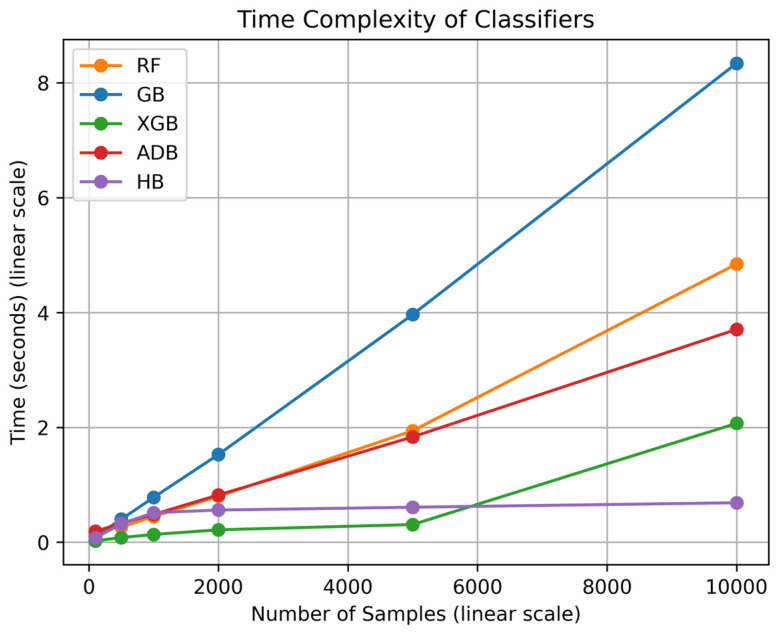
Time Complexity of Classifiers.

Figure 17 shows the Training Time Complexity curves for the ML models (classifiers) used in the study. The X-axis represents the number of samples (input dataset size) on a linear scale, while the Y-axis represents the training time in seconds, also on a linear scale. It can be observed that the curve for GB is the steepest, indicating that its training time increases sharply as the number of samples grows, making it a less time-efficient model. For instance, GB took more than 8 s to train on a dataset of 10,000 samples, which is considerably high relative to the other models.

In contrast, HB has the least steep curve, remaining mostly flat after a certain input size, indicating that HB is the most time-efficient model in the study. RF has the second steepest curve, making it the second least time-efficient model. XGB and ADB have curves that are steeper than HB but less steep than GB and RF, indicating that they are moderately time-efficient.

### 3.3. Interpretation Using Explainable AI

**Figure 18:**
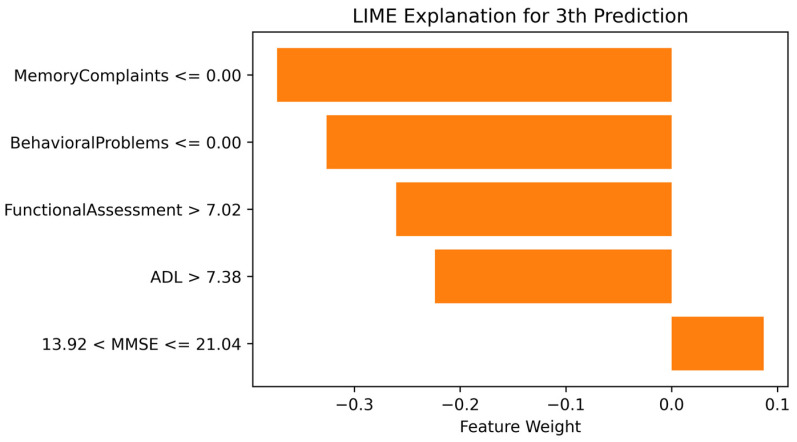
LIME explanation for the third instance predicted No Alzheimer’s Disease.

Figure 18 shows the LIME explanation for the third instance from the dataset. Memory Complaints, Behavioral Problems, FA score, and ADL score have negative weights, indicating they contributed to producing a negative output, i.e., no AD. In contrast, the MMSE score has a positive weight, contributing to producing a positive output, i.e., AD.

**Figure 19:**
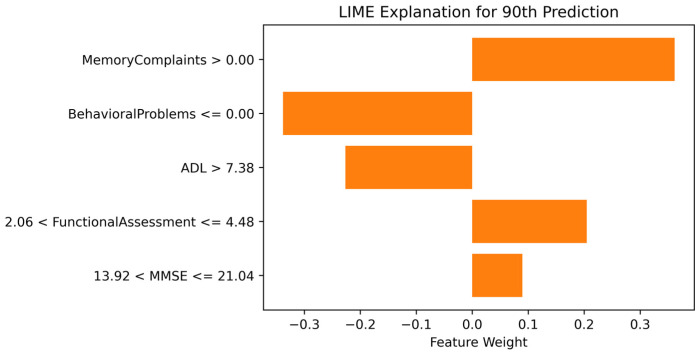
LIME explanation for the 90th instance predicted No Alzheimer’s Disease.

Figure 19 shows the LIME explanation for the 90th sample from the dataset. Memory Complaints, Functional Assessment (FA), and the Mini-Mental State Exam (MMSE) have positive weights, indicating they contributed to producing a positive output, i.e., Alzheimer’s Disease (AD). In contrast, Behavioral Problems and Activities of Daily Living (ADL) contributed to producing a negative output, i.e., no AD. Since LIME are local, this explanation may not fully align with the model’s overall behavior and is limited to this specific instance.

**Figure 20:**
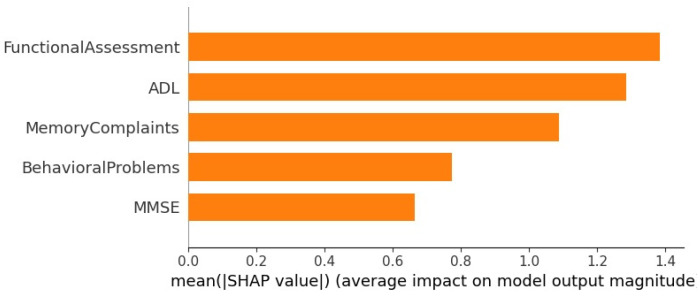
SHAP Explanation for Gradient Boosting Model.

Figure 20 shows the SHAP explanation for the Gradient Boosting model. It can be observed that, among the top five selected features, the Functional Assessment (FA) has the highest average impact on the model’s output. Conversely, the Mini-Mental State Exam (MMSE) score has the lowest average impact on the model’s output magnitude.

**Figure 21:**
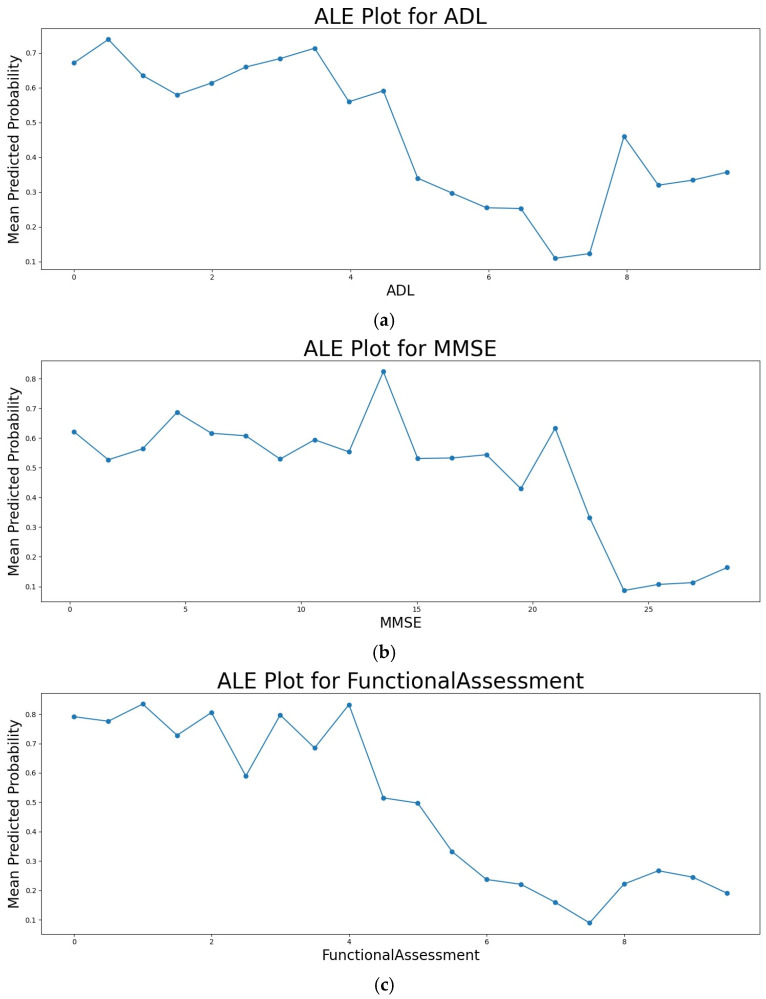
(**a**) ALE plot for Activities Daily Living Score (**b**) Functional Assessment Score (**c**) Mini Mental State Examination Score.

Figure 21a shows the ALE plot for the feature ADL score, illustrating how an increasing ADL score impacts the mean predicted probability of the AD class. It can be observed that as the ADL score increases, the mean predicted probability of the AD class generally decreases. This indicates that a higher ADL score influences the model to predict No AD. Figure 21b shows the ALE plot for the MMSE score. It illustrates how an increasing MMSE score impacts the mean predicted probability of the AD class. Although weak, a general downward trend can be observed, indicating that an increasing MMSE score also influences the model to predict No AD. The downward trend in Figure 21b is less steep than in Figure 21a, suggesting that the MMSE score has relatively less influence on the probability of the prediction being No AD compared to the ADL score. Figure 21c shows the ALE plot for the FA score. A steep downward trend can be observed, indicating that an increasing FA score causes the predicted probability of the AD class to decrease. The downward trend in Figure 21c is the steepest among the three, indicating that the FA score has the greatest impact on the predicted probability of the AD class.

Table 6 displays the weight (importance) of the features calculated using ELI5 on which the GB model is trained.

The third column contains the weights of the corresponding features. Each value in this column has two components: the estimated weight and the variability component. The estimated weight is the mean weight of the corresponding feature, while the variability component is the standard deviation, representing uncertainty. It can be observed that ELI5 has assigned the following weights: FA score, MMSE score, ADL score, Memory Complaints, and Behavioral Problems have weights of 0.2436 ± 0.3407, 0.2375 ± 0.3433, 0.2370 ± 0.4329, 0.1587 ± 0.1742, and 0.1232 ± 0.1544, respectively. The FA score has the highest mean weight of 0.2436 with a standard deviation of 0.3407. Having the highest mean weight indicates that the FA score is the most important feature among those considered. The MMSE score has the second-highest mean weight of 0.2375 and a standard deviation of 0.3433, making the MMSE score the second most important feature. The ADL score has a mean weight of 0.2370, slightly less than that of the MMSE score. Therefore, the MMSE score has almost equal importance to the ADL score. Behavioral Problems have the lowest mean weight, indicating it has the least importance among the selected features.

## 4. Discussion

The proposed framework for Alzheimer’s disease (AD) classification uses random sampling to handle data imbalance, exhaustive feature analysis to understand the influence and relationship of each feature with the diagnosis, dimensionality, and feature reduction, classification using boosting algorithms for diagnosis, and model interpretation using explainable AI. This approach proved to be effective, reliable, transparent, and computationally inexpensive compared to other similar methods. This section discusses the major contributions of the proposed framework, its implications for clinical practice, the improvements made, and the efficiency measures recorded.

### 4.1. Contribution

○ Addressing data imbalance through random sampling: Many existing studies in AD classification overlook the unequal distribution of disease stages, which can result in biased predictions and poor generalizability. By ensuring a more balanced representation of AD and No AD cases, the proposed model provides more reliable and equitable results.
○ An exhaustive feature analysis was conducted to investigate the magnitude and type of impact that demographic factors, lifestyle choices, medical history, clinical measurements, cognitive and functional assessments, and symptoms have on AD diagnosis.
○ Feature and Dimensionality Reduction: The K-best algorithm was used to select the 5 most impactful features, focusing the model on the key clinical indicators. This reduced dimensionality and enhanced the efficiency of the process. It was observed that the Functional Assessment Score, Mini-Mental State Examination Score, Activities of Daily Living Score, Memory Complaints, and Behavioral Problems were the most influential features.
○ Explainable AI techniques such as LIME, SHAP, ALE, and ELI5 were utilized to enhance the transparency of the results obtained for AD classification. Post-training, feature importance was observed for each selected feature, along with the type of influence these features had on the classification.
○ Ensemble Learning: Gradient Boosting (GB), XGBoost (XGB), AdaBoost (ADB), Histogram-based Gradient Boosting (HB), and Random Forest (RF) were used to develop the AD prediction model. Results showed that GB outperformed all other models, achieving the highest accuracy, precision, recall, and F1 score values of 95.18%, 95.20%, 95.18%, and 95.18%, respectively.
○ Training Time Complexity: The training times for each model on synthetic datasets of varying sizes were recorded and plotted. A comparative analysis of the 5 models revealed that GB was the least time-efficient as the dataset size increased, while HB emerged as the most time-efficient as the dataset size grew.

### 4.2. Implications for Clinical Practice

The proposed method, based on random sampling, feature analysis and reduction, ensemble learning, and explainable AI techniques, proves to be effective for clinical practice. Random sampling ensures the model is not biased towards any particular class. Extensive feature analysis determines the influence of each feature, allowing us to focus on the most important ones for model training. The use of boosting algorithms ensures quantitatively optimal results, making the approach superior. Incorporating LIME, SHAP, ALE, and ELI5 provides insights into feature importance post-training, enhancing the transparency and reliability of the predictions. Thus, this method can be considered for clinical applications.

### 4.3. Comparison with Existing Methods for AD Diagnosis

Table 7 illustrates a comparative analysis of the proposed method against other methods that use clinical or genetic data.

Kavitha et al. [45] used DT, RF, SVM, XGB, and a Voting Classifier on the clinical data, achieving accuracy, precision, recall, and F1 score values of 86.92%, 85%, 81%, and 80%, respectively. Khater et al. [46] employed SVM, RF, MLP, KNN, LightGBM, and ADB on the genetic data, generating accuracy, precision, recall, and F1 score values of 89% across the board, outperforming Kavitha et al. [45]. in every metric considered in Table 7. They also leveraged SHAP and LIME to interpret and explain the prediction models. Antor et al. used SVM, LR, DT, and RF on clinical data, producing superior accuracy, precision, recall, and F1 score values of 92%, 91.9%, 91.9%, and 91.9%, respectively. Almohimeed et al. [47] applied SVM, LR, KNN, NB, DT, RF, and a Stacking Model with SHAP analysis, resulting in accuracy, precision, recall, and F1 score values of 92.08%, 92.07%, 92.08%, and 92.01%, respectively, comparable to Antor et al. [44]. Our method produced accuracy, precision, recall, and F1 score values of 95.18%, 95.20%, 95.18%, and 95.18%, respectively, outperforming all other methods in comparison. We incorporated LIME, SHAP, ALE, and ELI5 to interpret the results of the prediction task. Thus, the proposed method successfully proves superior to other methods using clinical or genetic data.

## 5. Conclusions and Future Scope

### 5.1. Conclusion

This paper proposes a method to predict Alzheimer’s disease (AD) using ensemble-based models trained on a large dataset with various feature types, such as clinical measurements, symptoms, lifestyle factors, and demographic information. Random sampling is integrated to prevent the models from being biased towards a particular class. Feature analysis helped determine the magnitude and type of influence each feature had on the diagnosis. The K-best algorithm was employed to select the top five features, significantly reducing computational load and improving efficiency. The method was tested on five ensemble-based models: Gradient Boosting (GB), Histogram-based Gradient Boosting (HB), Adaptive Boosting (ADB), eXtreme Gradient Boosting (XGB), and Random Forest (RF). RF and XGB achieved the highest training accuracy (100%), but with testing accuracies of 94.96% and 93.20%, respectively. The large gap between training and testing accuracies indicates overfitting and poor generalization. HB achieved a training accuracy of 99.91% and a testing accuracy of 94.08%, indicating some degree of overfitting. ADB showed training and testing accuracies of 92.29% and 91.67%, respectively, suggesting no overfitting and decent generalizability. GB provided a solid training accuracy of 95.68% and an impressive testing accuracy of 95.18%, with an insignificant difference between the two values, indicating good generalization and resistance to overfitting. GB also exhibited the highest performance across various metrics, including testing accuracy, precision, recall, specificity, F1 score, Jaccard score, Dice coefficient, and Matthews correlation coefficient, among the five models considered. Explainable AI techniques such as LIME, SHAP, ALE, and ELI5 were employed to make predictions reliable and transparent by explaining the importance of each selected feature post-training. These explanations empower medical practitioners to understand the patterns influencing AD diagnosis. This study aims to improve the efficacy of AD prediction methods and enhance robustness.

### 5.2. Future Scope

Although the proposed method proves to be superior and reliable, there is room for improvement. Training and testing this method on different datasets, as well as combining diverse datasets from various geographies and with different feature types, will further enhance the process. Incorporating data collected over longer periods and with a wider range of feature values is also recommended. This would make the model more applicable to predicting AD in a larger population. Additionally, using a combination of Magnetic Resonance (MR) images and clinical data could further improve prediction performance. This could be achieved by employing two separate models—one for clinical data and another for image data—then combining their prediction results to obtain the final prediction. This approach would result in a more robust and diverse system.

## Figures and Tables

**Table 1:** Numerical features.

SN	Feature Categories	Features	Range	Mean Value	Correlation
1	Demographic	Age	60–90	75	0.005
2	Lifestyle Factors	Body Mass Index	15–40	28	0.026
3		Alcohol Consumption (in units)	0–20	10	0.007
4		Weekly Physical Activity (in hours)	0–10	5	0.0059
5		Diet Quality Score	0–10	5	0.0085
6		Sleep Quality score	4–10	7	0.057
7	Clinical Measurements	Systolic BP (in mmHg)	90–180	135	0.016
8		Diastolic BP (in mmHg)	60–120	90	0.005
9		Cholesterol Total (in mg/dL)	150–300	225	0.006
10		Cholesterol LDL (in mg/dL)	50–200	125	0.032
11		Cholesterol HDL (in mg/dL)	20–100	60	0.043
12		Cholesterol Triglycerides (in mg/dL)	50–400	228	0.023
13	Cognitive and Functional Assessments	Mini-Mental State Examination Score	0–30	15	−0.24
14		Functional Assessment	0–10	5	−0.36
15		Activities of Daily Living Score	0–10	5	−0.33

**Table 2:** Categorical features.

SN	Feature Categories	Features	Category	Correlation
1	Demographic	Gender	0 (Male) 1 (Female)	0.021
2		Ethnicity	0: Caucasian 1: African American 2: Asian 3: Other	0.015
3		Education Level	0: None 1: High School 2: Bachelor’s 3: Higher	0.044
4	Lifestyle Factors	Smoking	0 (No) and 1 (Yes)	0.004
5	Medical History	Family History	0 (No) and 1 (Yes)	0.033
6		Cardiovascular Disease	0 (No) and 1 (Yes)	0.031
7		Diabetes	0 (No) and 1 (Yes)	0.032
8		Depression	0 (No) and 1 (Yes)	0.005
9		Head Injury	0 (No) and 1 (Yes)	0.023
10		Hypertension	0 (No) and 1 (Yes)	0.035
11	Cognitive and Functional Assessments	Memory Complaints	0 (No) and 1 (Yes)	0.31
12		Behavioral Problems	0 (No) and 1 (Yes)	0.22
13	Symptoms	Confusion	0 (No) and 1 (Yes)	−0.019
14		Disorientation	0 (No) and 1 (Yes)	0.025
15		Personality Changes	0 (No) and 1 (Yes)	0.021
16		Difficulty Completing Tasks	0 (No) and 1 (Yes)	0.009
17		Forgetfulness	0 (No) and 1 (Yes)	0.0003

**Table 3:** Performance comparison of ML models based on their accuracy.

Model	Training Accuracy	Testing Accuracy
Random Forest	1	0.949561
Gradient Boosting	0.956767	0.951754
eXtreme Gradient Boosting	1	0.932018
Adaptive Boosting	0.922932	0.916667
Histogram Gradient Boosting	0.99906	0.940789

**Table 4:** Quantitative evaluation of ML models based on Precision, Recall, Specificity, and F1 Scores.

Model	Precision	Recall	Specificity	F1 Score
Random Forest	0.949704	0.949561	0.948	0.949591
Gradient Boosting	0.951976	0.951754	0.948	0.951792
eXtreme Gradient Boosting	0.932953	0.932018	0.92	0.932121
Adaptive Boosting	0.921322	0.916667	0.88	0.916861
Histogram Gradient Boosting	0.941129	0.940789	0.936	0.940845

**Table 5:** Quantitative evaluation of ML models based on Jaccard Score, Dice Coefficient, and Matthews Correlation Coefficient.

Model	Jaccard Score	Dice Coefficient	Matthews Correlation Coefficient
Random Forest	0.894977	0.944578	0.898384
Gradient Boosting	0.899543	0.947115	0.902909
eXtreme Gradient Boosting	0.862832	0.926366	0.863964
Adaptive Boosting	0.838983	0.912442	0.83724
Histogram Gradient Boosting	0.878378	0.935252	0.880936

**Table 6:** Weights of the features with impact on AD diagnosis using ELI5.

Sr. No.	Feature	Weight
1.	Functional Assessment	0.2436 ± 0.3407
2.	MMSE	0.2375 ± 0.3433
3.	ADL	0.2370 ± 0.4329
4.	Memory Complaints	0.1587 ± 0.1742
5.	Behavioral Problems	0.1232 ± 0.1544

**Table 7:** Comparative analysis of the results obtained for AD diagnosis using the proposed framework with existing methods.

Sr. No	Method	Dataset	Classifiers	Best Evaluation	Explainable AI
1.	Antor et al. [44]	Clinical Data	SVM, LR, DT, RF	Accuracy: 92.0% Precision: 91.9% Recall: 91.9% F1-Score: 91.9%	-
2.	Kavitha et al. [45]	Clinical Data	DT, RF, SVM, XGB, Voting Classifier	Accuracy: 86.92% Precision: 85% Recall: 81% F1-Score: 80%	-
3.	Khater et al. [46]	Genetic Data	SVM, RF, MLP, KNN, LightGBM, ADB	Accuracy: 89% Precision: 89% Recall: 89% F1-Score: 89%	LIME and SHAP
4.	Almohimeed et al. [47]	Clinical Data	SVM, LR, KNN, NB, DT, RF, Stacking Model	Accuracy: 92.08% Precision: 92.07% Recall: 92.08% F1-Score: 92.01%	SHAP
5.	Proposed Method	Clinical Data	RF, HB, GB, XGB and ADB	Accuracy: 95.18% Precision: 95.20% Recall: 95.18% F1-Score: 95.18%	LIME, SHAP, ALE and ELI5

## Data Availability

Data are publicly available on the Kaggle Website https://www.kaggle.com/datasets/rabieelkharoua/alzheimers-disease-dataset, accessed on 1 September 2024.

## References

[1] Huang L.K., Kuan Y.C., Lin H.W., Hu C.J. (2023). Clinical trials of new drugs for Alzheimer disease: A 2020–2023 update. J. Biomed. Sci..

[2] Arya A.D., Verma S.S., Chakarabarti P., Chakrabarti T., Elngar A.A., Kamali A.M., Nami M. (2023). A systematic review on machine learning and deep learning techniques in the effective diagnosis of Alzheimer’s disease. Brain Inform..

[3] Alshamlan H., Omar S., Aljurayyad R., Alabduljabbar R. (2023). Identifying effective feature selection methods for Alzheimer’s disease biomarker gene detection using machine learning. Diagnostics.

[4] Alamro H., Thafar M.A., Albaradei S., Gojobori T., Essack M., Gao X. (2023). Exploiting machine learning models to identify novel Alzheimer’s disease biomarkers and potential targets. Sci. Rep..

[5] Rajab M.D., Jammeh E., Taketa T., Brayne C., Matthews F.E., Su L., Cognitive Function and Ageing Neuropathology Study Group (2023). Assessment of Alzheimer-related pathologies of dementia using machine learning feature selection. Alzheimer’s Res. Ther..

[6] Sudharsan M., Thailambal G. (2023). Alzheimer’s disease prediction using machine learning techniques and principal component analysis (PCA). Mater. Today Proc..

[7] Lmatrafi S., Abbas QIbrahim M.E.A. (2024). A systematic literature review of machine learning approaches for class-wise recognition of Alzheimer’s disease using neuroimaging-based brain disorder analysis. Multimed Tools Appl..

[8] Patil V., Madgi MKiran A. (2022). Early prediction of Alzheimer’s disease using convolutional neural network: A review. Egypt. J. Neurol. Psychiatry Neurosurg..

[9] Friedman J.H. (2001). Greedy function approximation: A gradient boosting machine. Ann. Stat..

[10] Chen T., Guestrin C. XGBoost: A scalable tree boosting system. Proceedings of the 22nd ACM SIGKDD International Conference on Knowledge Discovery and Data Mining.

[11] Ke G., Meng Q., Finley T., Wang T., Chen W., Ma W., Ye Q., Liu T. LightGBM: A highly efficient gradient boosting decision tree. Proceedings of the 31st International Conference on Neural Information Processing Systems (NeurIPS).

[12] Hastie T.J., Rosset S., Zhu J., Zou H. (2009). Multi-class AdaBoost. Stat. Its Interface.

[13] Freund Y., Schapire R.E. (1997). A decision-theoretic generalization of on-line learning and an application to boosting. J. Comput. Syst. Sci..

[14] Benbouzid D., Busa-Fekete R., Casati N., Hsu W.H., Kégl B. (2012). MultiBoost: A multi-purpose boosting package. J. Mach. Learn. Res..

[15] Luo P., Xiong Y., Liu W., Tang X. Efficient mini-batch training for stochastic gradient boosting decision trees. Proceedings of the 22nd ACM SIGKDD International Conference on Knowledge Discovery and Data Mining.

[16] Breiman L. (2001). Random forests. Mach. Learn..

[17] Liaw A., Wiener M. (2002). Classification and regression by randomForest. R News.

[18] Kaur A., Mittal M., Bhatti J.S., Thareja S., Singh S. (2024). A systematic literature review on the significance of deep learning and machine learning in predicting Alzheimer’s disease. Artif. Intell. Med..

[19] Alatrany A.S., Khan W., Hussain A., Kolivand H., Al-Jumeily D. (2024). An explainable machine learning approach for Alzheimer’s disease classification. Sci. Rep..

[20] Guidotti R., Monreale A., Ruggieri S., Turini F., Giannotti F., Pedreschi D. (2018). A survey of methods for explaining black box models. ACM Comput. Surv. (CSUR).

[21] Molnar C. Interpretable Machine Learning: A Guide for Making Black Box Models Explainable. https://christophm.github.io/interpretable-ml-book/.

[22] Tjoa E., Guan C. (2021). A Survey on Explainable Artificial Intelligence (XAI): Toward Medical XAI. IEEE Trans. Neural Netw. Learn. Syst..

[23] Linardatos P., Papastefanopoulos V., Kotsiantis S. (2021). Explainable AI: A Review of Machine Learning Interpretability Methods. Entropy.

[24] Yi F., Yang H., Chen D., Qin Y., Han H., Cui J., Bai W., Ma Y., Zhang R., Yu H. (2023). XGBoost-SHAP-based interpretable diagnostic framework for alzheimer’s disease. BMC Med. Inform. Decis. Mak..

[25] Dutta M., Hasan K.M.M., Akter A., Rahman M.H., Assaduzzaman M. (2024). An interpretable machine learning-based breast cancer classification using XGBoost, SHAP, and LIME. Bull. Electr. Eng. Inform..

[26] Thakur A., Arunbalaji C.G., Maddi A., Maheswari B.U. Interpretable Predictive Modeling for Smoking and Drinking Behavior using SHAP and LIME. Proceedings of the 2024 International Conference on Current Trends in Advanced Computing (ICCTAC).

[27] Aydin H.E., Iban M.C. (2023). Predicting and analyzing flood susceptibility using boosting-based ensemble machine learning algorithms with SHapley Additive exPlanations. Nat. Hazards.

[28] Ekanayake I.U., Meddage D.P.P., Rathnayake U. (2022). A novel approach to explain the black-box nature of machine learning in compressive strength predictions of concrete using Shapley additive explanations (SHAP). Case Stud. Constr. Mater..

[29] Sahlaoui H., Alaoui E.A.A., Agoujil S., Nayyar A. (2024). An empirical assessment of smote variants techniques and interpretation methods in improving the accuracy and the interpretability of student performance models. Educ. Inf. Technol..

[30] Ma X., Hou M., Zhan J., Liu Z. (2023). Interpretable predictive modeling of tight gas well productivity with SHAP and LIME techniques. Energies.

[31] El Bilali A., Abdeslam T., Ayoub N., Lamane H., Ezzaouini M.A., Elbeltagi A. (2023). An interpretable machine learning approach based on DNN, SVR, Extra Tree, and XGBoost models for predicting daily pan evaporation. J. Environ. Manag..

[32] Vimbi V., Shaffi N., Mahmud M. (2024). Interpreting artificial intelligence models: A systematic review on the application of LIME and SHAP in Alzheimer’s disease detection. Brain Inform..

[33] Ribeiro M.T., Singh S., Guestrin C. “Why should I trust you?”: Explaining the predictions of any classifier. Proceedings of the 22nd ACM SIGKDD International Conference on Knowledge Discovery and Data Mining.

[34] Ribeiro M.T., Singh S., Guestrin C. Anchors: High-precision model-agnostic explanations. Proceedings of the AAAI Conference on Artificial Intelligence.

[35] Arrieta A.B., Díaz-Rodríguez N., Del Ser J., Bennetot A., Tabik S., Barbado A., Garcia S., Gil-Lopez S., Molina D., Benjamins R. (2020). Explainable Artificial Intelligence (XAI): Concepts, taxonomies, opportunities and challenges toward responsible AI. Inf. Fusion.

[36] Islam M., Zhang Y., Venkatesh S., Mahmud M., Venkatesh S. Interpretable machine learning models for Alzheimer’s disease classification with LIME feature importance analysis. Proceedings of the 2020 International Joint Conference on Neural Networks (IJCNN).

[37] Lundberg S. (2017). A unified approach to interpreting model predictions. arXiv.

[38] Lundberg S.M., Erion G.G., Chen H., DeGrave A., Prutkin J.M., Nair B., Katz R., Himmelfarb J., Bansal N., Lee S.I. (2020). From local explanations to global understanding with explainable AI for trees. Nat. Mach. Intell..

[39] Liu F.X. (2024). Evaluating the Impact of Data Augmentation on Explainable AI in Medical Image Analysis. Master’s Thesis.

[40] Rabiee M., Mirhashemi M., Pangburn M.S., Piri S., Delen D. (2024). Towards explainable artificial intelligence through expert-augmented supervised feature selection. Decis. Support Syst..

[41] Apley D.W., Zhu J. (2020). Visualizing the Effects of Predictor Variables in Black Box Supervised Learning Models. J. R. Stat. Soc. Ser. B Stat. Methodol..

[42] Ghahraman V., Tamimy M. (2021). Conversation analysis: An ELI5 overview. Int. J. Lang. Stud..

[43] https://www.kaggle.com/datasets/rabieelkharoua/alzheimers-disease-dataset.

[44] Antor M.B., Jamil A., Mamtaz M., Khan M.M., Aljahdali S., Kaur M., Singh P., Masud M. (2021). A comparative analysis of machine learning algorithms to predict Alzheimer’s disease. J. Healthc. Eng..

[45] Kavitha C., Mani V., Srividhya S.R., Khalaf O.I., Romero C.A.T. (2022). Early-stage Alzheimer’s disease prediction using machine learning models. Front. Public Health.

[46] Khater T., Abdelhalim A.M., Tolba E.M., Hassanien A.E. (2024). Explainable Machine Learning Model for Alzheimer Detection Using Genetic Data: A Genome-Wide Association Study Approach. IEEE Access.

[47] AlMohimeed A., Saad R.M.A., Mostafa S., El-Rashidy N.M., Farrag S., Gaballah A. (2023). Explainable Artificial Intelligence of Multi-Level Stacking Ensemble for Detection of Alzheimer’s Disease Based on Particle Swarm Optimization and the Sub-Scores of Cognitive Biomarkers. IEEE Access.

